# Patient, caregiver, health professional and researcher views and experiences of participating in research at the end of life: a critical interpretive synthesis of the literature

**DOI:** 10.1186/1471-2288-12-123

**Published:** 2012-08-17

**Authors:** Marjolein H Gysels, Catherine Evans, Irene J Higginson

**Affiliations:** 1 King’s College London, Cicely Saunders Institute, Department of Palliative Care, Policy & Rehabilitation School of Medicine, London, UK

**Keywords:** Research participation, End of life, Palliative care, Evidence, Critical interpretive synthesis

## Abstract

**Background:**

The development of the evidence-base informing end of life (EoL) care is hampered by the assumption that patients at the EoL are too vulnerable to participate in research. This study aims to systematically and critically review the evidence regarding the experiences and views of patients, caregivers, professionals and researchers about participation in EoL care research, and to identify best practices in research participation.

**Methods:**

We searched seven electronic databases, and hand searched three journals and the bibliographies of relevant papers. Inclusion criteria were original research papers on involvement in EoL care research or its impact on participants. Critical interpretive synthesis was used to integrate the whole body of empirical evidence on this topic and generate theoretical categories from the evidence.

**Results:**

Of a total of 239 identified studies, 20 studies met the inclusion criteria, from: the US (11), the UK (6) and Australia (3). Most focused on patients with cancer (12) and were conducted in hospices (9) or hospitals (7). Studies enquired about issues related to: EoL care research in general (5), specific research methods (13), and trial research (2). The studies evaluating willingness to participate in EoL care research showed positive outcomes across the different parties involved in research. Factors influencing willingness were mainly physical and cognitive impairment. Participating in research was a positive experience for most patients and carers but a minority experienced distress. This was related to: characteristics of the participants; the type of research; or the way it was conducted. Participatory study designs were found particularly suitable for enabling the inclusion of a wide range of participants.

**Conclusion:**

The evidence explored within this study demonstrates that the ethical concerns regarding patient participation in EoL care research are often unjustified. However, research studies in EoL care require careful design and execution that incorporates sensitivity to participants’ needs and concerns to enable their participation. An innovative conceptual model for research participation relevant for potentially vulnerable people was developed.

## Background

According to projections, the number of people aged 65 and above will increase dramatically by 2030 (one in eight of the global population) [1]. A demographic shift is occurring, with people generally living longer and a smaller number dying from infectious diseases [2]. This brings a different and currently not well-understood scenario - involving older people suffering from chronic illnesses with complex and clinically challenging trajectories. This rapidly changing situation will create substantially greater end of life (EoL) care needs [3].

Within this paper, EoL care is understood in its broadest sense, referring to the care provided to people with advanced, progressing and incurable disease and their families. It covers the therapeutic approaches from diagnosis on, as well as the care provided at the very end of life, during death and the bereavement process.

Deficiencies in EoL care have been reported, such as in the area of pain and symptom management [3]. There is a need for research involving patients at the end of their lives in order to develop and test new treatments and services and to ensure the provision of high-quality and cost-effective care [4-7]. Numerous practical and ethical obstacles to research during the EoL are well documented [8,9]. Trial research is often hampered by problems of recruitment, high attrition rates, missing data and a lack of outcome measures validated with palliative care patients [10].

The ethical arguments between the value of research and the needs of patients are heavily debated and complex [11].

The debate reflects a community of stakeholders that is polarised between those who argue that research within this population is unjustified and those who argue that the ethical principles that govern medical research are also suitable to safeguard individuals’ interests to enable their participation in EoL care research. While it is necessary to consider the concerns of this debate there is little prospect of resolving the existing tensions by argumentation alone. Systematic reviews have appraised the evidence regarding advanced cancer patients’ attitudes towards research [12] and on palliative care patients’ and carers’ views about research in palliative care [13]. This study broadens the scope by undertaking a critical interpretive synthesis (CIS) [14] across this field in order to synthesise existing empirical data on the attitudes and values regarding participation held by all stakeholders involved in palliative care research. Attention has been given to the methodological issues that emerge from these studies.

### Aim

The study aims to appraise the evidence regarding all stakeholders’ views and experiences regarding involvement in palliative care research and to explore whether there are best practices regarding participation in research.

### Research questions

· Which stakeholders’ views on participation in research are represented in the evidence and what are the views held?

· Are patients and other stakeholders willing to participate in EoL care research?

· How is participation in EoL care research experienced?

· Are there differences in attitude according to group (patient or other participants’ groups)?

· What are the best practices to enable patients’ participation in research on EoL care?

## Methods

### Design

We used CIS [14] to synthesise a diverse and complex body of evidence consisting of both quantitative and qualitative studies. This methodology incorporates procedures of conventional systematic review methodology by applying explicit searching strategies to ensure replicable, specified inclusion criteria, and data extraction procedures to enhance clarity and comparability among studies. This was combined with recent methods for interpretive synthesis which drew on traditions of qualitative enquiry. This combined approach enabled both aggregation of the literature and the generation of theoretical categories that critically demonstrate the assumptions situated within the literature.

### Search strategy

The search was conducted in four electronic databases: MEDLINE (1950 – January week 2 2010); EMBASE (1980 – January week 2 2010); PsycINFO (1806 – January week 2 2010); and CINAHL (1982 – January). Additionally the following databases were searched for relevant articles: Cochrane Database of Systematic Reviews, The Cochrane Central Register of Controlled Trials, Cochrane Pain, Palliative and Supportive Care Trials Register.

The combination of search terms was piloted with a number of trial searches to achieve a balance between recall and specificity. A pre-defined search strategy was decided upon, consisting of the following search terms, representing three groups which were combined with AND.

1. attitude$, OR view$, OR experience$, involv$, partic$, perspect$, concern$, challeng$

2. research (Focus)

3. palliative care, OR palliat$, OR terminal, OR terminally ill, OR supportive care, OR dying, OR death, OR end of life, OR end-of-life, OR hospice, OR bereavement.

We searched the reference lists of all relevant papers for further references. This was important as this topic concerns a body of evidence, which is not well delineated and is spread over the disparate research areas that address palliative care.

Three journals, in which the debate regarding whether or not to involve palliative care patients in research was conducted, were hand searched. These were:

· The Journal of Pain and Symptom Management (from 1999 to February 2010)

· Palliative Medicine (from 1989 to February 2010)

· Journal of Palliative Care (from 1985 to June 2010)

### Inclusion and exclusion criteria

We included original research papers that enquired specifically about involvement in EoL care research or its impact on participants. The participants included a wide variety of stakeholders who come into contact with palliative care research, including: patients, informal caregivers, health professionals, managers, and researchers. We included all research designs and searched across the spectrum of palliative care from disease diagnosis to death.

We excluded studies on oncology patients participating in trials testing curative treatments. We excluded papers on children or adults with learning disabilities as these areas have their own specific issues; and studies on recruitment, informed consent, user involvement or bereavement. Non-English language papers were excluded. We did not include papers that were already included in previous reviews. However, we investigated the individual studies in these reviews and included them if they contributed to our review questions and responded to our quality criteria. Fatally flawed studies were excluded. To identify such low-quality studies we used the following criteria (as specified in [14]):

· Are the aims and objectives of the research clearly stated?

· Is the research design clearly specified and appropriate for the aims and objectives?

· Do researchers provide a clear account of the process by which their findings were produced?

· Do the researchers display enough data to support their interpretations and conclusions?

· Is the method of analysis appropriate and adequately explicated?

### Synthesis

First, the identified studies were mapped according to country of origin, setting, participants, patient group, illness stage, and approach. The resultant studies were then counted. Three major categories were identified: studies that addressed issues relating to research in general; research with specific methods; and trials. Within these categories, studies were examined in more detail and the results compared. This revealed areas that were adequately covered and any remaining gaps.

In the second stage the research questions were applied to the literature across the categories. Results and emerging queries were listed. Recommendations from the studies were tabulated and organised inductively, thereby generating information on best practices.

A third stage focused on the normative assumptions underlying the studies and related these to the empirical evidence they generated. The characteristics attributed to ‘research’ and ‘care’ – and how these determined their conceptualization – were critically assessed. Whether the interpretation changed with each included study was continually tested. The findings are synthesised as a conceptual model for research participation for potentially vulnerable people.

## Results

### Flow chart of search results

**Figure 1:**
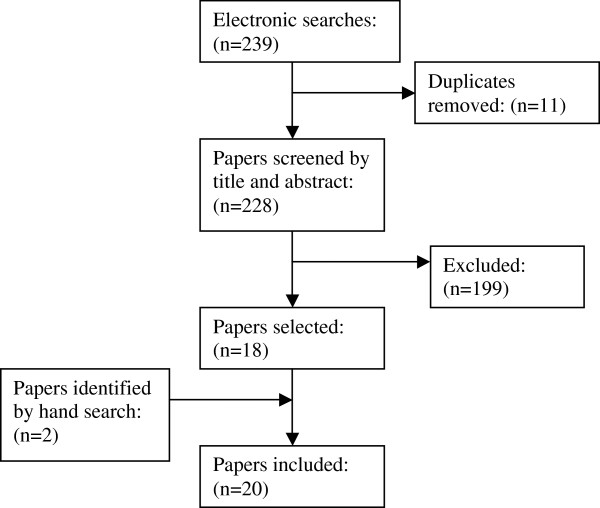
Flow chart.

Please include Figure 1

### The nature of the evidence

The characteristics of the studies are summarized in Table 1 and Table 2. The evidence generated was of three different types. Most studies (14) based their findings on individuals’ directly reported experiences of participating themselves in a research study. The other studies approached the topic indirectly by enquiring about attitudes [15,16], by enquiring about participants’ settled opinions or ways of thinking about research; or asking hypothetical questions [17-20] to respondents where they are asked about a supposed situation where people who receive palliative care are invited to participate or are participating in research. These categories are denoted in Table 2 as D (direct), A (attitudinal), and H (hypothetical). 

**Table 1:** The nature of the evidence

**Study country**	
USA	11
UK	6
Australia	3
Study setting	
Hospices	9
Hospice at home	1
Inpatient hospice and patients from the community	1
Hospital	7
Home	1
Setting not clear	1
Participants	
Patients	12
Health professionals	3
Carers	1
Researchers	1
Mixed	2
Managers	1
Diagnosis	
Cancer	12
Not specified	5
Mixed	2
Not applicable	1
Stage	
Advanced	13
Terminal	5
Early	1
Not applicable	1

**Table 2:** Research participation according to type of research

**Study details**	**Type of research Objectives**	**Study design**	Assessment: *D,H, A	**Setting**	**Participants**	**Method of evaluation**	**Main outcome**
**Research in general**							
[16]	To define the research that is conducted in hospices across the US, assess perceptions of the obstacles to research, and define their views about the types of that would be important to them	Nationwide telephone survey	A	Hospice	Organisations	Prospective	Hospice staff willing to support research on: Pain management, and referral to hospice
[21]	To describe the experiences in establishing a designated social work research unit in a major academic cancer research institution	Social work research unit in academic cancer hospital	D	Hospital	Researchers/clinicians	Retrospective	Researchers and clinicians developed appreciation for each other’s work and limitations, in turn facilitating teamwork
[22]	Evaluation of data registry of patients willing to participate in psychosocial cancer research	Participation involved completing a standard data set of psychosocial measures and agreeing to future follow-up contacts and data collection at three months and at 12 months post-enrollment	D	Hospital	Cancer patients at diagnosis	Retrospective	The majority of newly diagnosed patients approached for consent (68%) and their primary family caregiver (92%) were willing to participate in the registry; of these, 80% also agreed to be contacted in the future for additional studies. Face-to-face interview was the preferred method of data collection. The majority (about 70%) of newly diagnosed adults with cancer participated in a data registry
[15]	To examine hospice staff attitudes, beliefs, values about research with their patients, and family	Cross-sectional, anonymous survey	A	Hospice	Staff	Prospective	Staff were largely supportive of research with patients and families (mean agreement of 4.08-4.44). They acknowledged a mixture of being protective (52% wanted to approach the patients) and not having enough time for research (59% had no time or were willing to spend no more than 10 minutes on research)
[18]	To explore hypothetical interest in research participation amongst hospice patients and carers, compared to ambulatory senior citizens	Cross-sectional survey to explore interest in survey and therapeutic research	H	Hospice	Patients and carers. Diagnosis not specified Compared with senior ambulatory citizens	Prospective	Hospice patients and carers were interested: 46% and 60% respectively) and in therapeutic research (45% and 57% respectively). Younger hospice patients were more likely to be interested than older people. No difference was found in older hospice and non-hospice patients
**Research using surveys, interviews, observational and participatory methods**							
[23]	To investigate the potential of causing distress with interview-based research	Patients participated in a one-hour interview in relation to their medical care. They provided feedback regarding the interview itself	D	Home	Patients with terminal cancer	Retrospective add-on	Participation can be a positive, therapeutic experience
[24]	To reflect retrospectively on the ethical aspects of a study on patients with malignant cerebral glioma, and the effect of research on management	Experience of qualitative structured interviews with cancer patients	D	Hospital	Patients with advanced cancer	Retrospective add-on	63% gained comfort from being interviewed. 5% did not like the experience. There was a 95% follow up rate of the interviews. Feasible to interview patients/carers separately
[25]	To describe issues and dilemmas related to nonparticipation, attrition, and the need for assistance in research with vulnerable home hospice participants	Mixed methods study with home hospice patients with mixed diagnoses. Retrospective analysis, with descriptive statistics of the frequency of issues and dilemmas that occurred in a research study with a vulnerable population	D	Hospice	Patients, diagnosis not specified	Retrospective	From 113 potential participants, 16 (14.1%) people who gave initial consent were unable to participate or were lost to the study (subset I) for reasons: unable to give informed consent, cognitive disturbance, and physical distress. Of the 97 participants who completed testing, 28 (28.8%) required assistance (subset II) because of physical impairments. Those who completed testing expressed gratitude at being able to contribute information that they believed would benefit others.
[26]	Interviews and interviewed again two to six months later. Carers were interviewed separately	Interviews terminally ill patients and carers about death, dying and bereavement: stressful and/or helpful	D	Hospital	Patients with advanced illness (not specified) and carers	Retrospective add-on	87% of approached patients and 97% of carers were interviewed. *Patients’ distress:* 1.9% reported a great deal, 7.1% some, and 88% little or no stress from the interview. *Carers’ distress:* 1.5% reported a great deal, 8.4% some, 89% little or no stress. Slightly more stress at re-interview. *Patients finding interview helpful:* 16% very, 29% somewhat, 49% little or not. *Carers finding interview helpful:* 19% very, 34 somewhat, 44% little or not. The second interview was seen as slightlyless helpful. People with pain, more personal meaning in dying and less at ease with talking about dying reported more stress. Patients from ethnic minorities, anxious about EoL, spiritual, serene found interview helpful
[27]	To explore patients’ and carers’ preferences and expectations regarding their contribution to research in palliative care	Qualitative interviews were conducted in the context of two studies on the experiences of care by patients and carers	D	Hospital and community	Patients and carers with cancer, COPD, heart failure and MND	Retrospective add-on	Differential patterns in decline and acceptance of interviews by patients with different conditions and across settings were found. Among cancer patients, 21/51 declined; the People affected by cancer and researchers suggested that many people nearing the end of life do want to be offered the chance to participate in research, provided it is conducted sensitively. Although such research can be demanding, most researchers believed it to be no more problematic than many other areas of research and that the challenges identified can be overcome
[28]	To understand key challenges in researching EoL issues and identify ways of overcoming these	Qualitative study involving in-depth interviews with researchers and focus groups with people affected by cancer	D	Hospice and community	An international sample of 32 researchers; seven patients with experience of cancer; and four carers	Prospective	People affected by cancer and researchers suggested that many people nearing the end of life do want to be offered the chance to participate in research, provided it is conducted sensitively. Although such research can be demanding, most researchers believed it to be no more problematic than many other areas of research and that the challenges identified can be overcome
[29]	To assess the burden and benefits of participation in psychosocial research addressing EoL issues among patients receiving inpatient palliative care	Psychosocial Interview + Selfreport QNN.	D	Hospital	Patients with terminal cancer	Retrospective add-on	For 75% no burden, 68% moderately to highly beneficial. Most distressing: discuss death
[30]	To compare consenting advanced cancer patient participants and refusers in observational EoL research	Reasons for refusal to an observational study were recorded verbatim and coded using qualitative techniques	D	Not specified	Patients with advanced cancer	Retrospective add-on	Consenters believed that they had more to gain from participation. Consenters felt that *aches or pain* were more a problem
[20]	To explore the views and preferences of patients with advanced cancer on taking part in planned longitudinal questionnaire-based research studies	Semi-structured interviews in a study with cross-sectional design	H	Hospital	Patients with advanced cancer	Prospective	Preferred health professional known to them to contact them about study. Face-to face interviews were preferred. Flexibility in planning interviews is important. Patients preferred to be interviewed at home but were prepared to take part in hospital. Fluctuating symptom control needs may affect ability and willingness
[31]	To determine whether caregivers believe that interviews about EoL care are distressing and identify patient and respondent characteristics associated with an increased risk of distress	Four studies approaching families interviews Study 1: mailed survey Study 2,3,4: telephone survey	D	Hospice	Carers of patients with different diagnoses in advance d illness	Retrospective add-on	Minority reported distress, which was mild or moderate Higher risk: younger, cancer, women
[19]	To investigate whether the concerns researchers have about including terminally ill patients in research were shared by a sample of terminally ill patients	A descriptive qualitative analysis of the views of hospice patients on the problems of carrying out research with dying patients	H	Hospice	Terminally ill patients	Prospective	All the patients wanted to participate in research. Patients advanced one or more of several reasons for participation, the commonest being altruism, enhancement of a sense of personal value, the assertion of persisting autonomy and the value they placed on a commitment by doctors to optimising care by research. They rejected the view that their consent might be non-autonomous and put forward consistent views about what they considered relevant to consent
[32]	Experience of working with co-researchers to collect data from two hospices	The Macmillan Listening Study, a UK-wide study exploring research views and priorities of people affected by cancer, adopted a participatory research approach. A total of 17 focus groups were held.	D	Hospice	Patients as coresearchers	Retrospective	No-one was distressed Worried to ask inappropriate questions. Role researcher-participant blurred. Not possible to involvement in complete process.
[33]	To illustrate the use of qualitative methods in thanatology	Descriptive study of naturalistic enquiry.	D	Hospice	Patients with advanced illness and researcher	Retrospective	They assess risk and benefits: patients experiencing pain from discussing sensitive issues. Patients can sometimes open up more easily to researchers than health professionals. Researcher experienced difficulties with access to patients. The researcher reflects on the impact of this research on herself, recognising sadness but also meaningfulness
**Trial research**							
[34]	To investigate the impact of a three-year RCT of different models of service provision on staff	A qualitative substudy to a large three-year RCT of different models of service provision on staff	D	Hospice	Palliative care staff	Prospective	Initially the impact of the trial produced high levels of staff stress, diminishing largely over time, to be replaced by enthusiasm for the changes achieved and sadness that on trial completion the perceived benefits gained would be lost
[17]	To determine if patients with advanced cancer are interested in participation in research that does not involve anti-cancer therapy, particularly in the context of a RCT, and if so, what factors are important in their decisions. What level of inconvenience is tolerable and by which factors is willingness to participate influenced?	Self-administered questionnaires	H	Hospital	Patients with advanced cancer and relatives	Prospective	Patients with advanced cancer are interested in participating in RCTs focusing on symptom control. Relatives are supportive of participation. Over 75% expressed altruistic views. Many were prepared to complete short questionnaires, accept extra medications, investigations, hospital visits or admissions within a trial context. *Deterrants*: concepts of ‘randomisation’, ‘placebo-control’ and ‘blinding’, and the possibility of side effects. Patients’ age was the only significant predictor of willingness to participate

*Direct (D).

Hypothetical (H).

Attitudes (A).

All the studies that enquired about attitudes to research participation, or hypothetical research participation used prospective designs (6). The studies that examined experiences of research participation used a retrospective design (14). They either employed descriptions of interventions or studies; analysed data recording the research process (6) [21,22,25,32-34]; or a study was nested within the primary study to examine participants’ experiences of the research process (8) [23,24,26-31].

### Types of studies and their findings

Five studies considered participation in palliative care research in general. Eleven studies enquired about issues related to specific research methods that included questionnaires, qualitative interviews, focus groups, or observational and participatory methods. One study reported on feedback to an observational design from epidemiology or the social sciences [20]. Two studies assessed the views of those involved in clinical trial research [17,34].

#### Participation in research in general

The studies that assessed participation in research in general reported willingness towards participation mostly from hospice environments. Cassarett et al. reported on a nation-wide telephone survey to define the research that was conducted in hospices across the US, to assess hospice organisations’ perceptions regarding obstacles to research, and to identify their research priorities. They found that participation in research was related to hospice characteristics: urban location, academic affiliation, larger census and having an inpatient unit. This raises concerns about the generalisability of these results [16]. Kirsch et al. conducted a cross-sectional anonymous survey with hospice staff to gain insight into staff’s attitudes, beliefs and values about research with the patients they cared for and their families. They found that staff were largely supportive of research, but they acknowledged a mix of protectionism towards patients and not having enough time for research as barriers to participation [15]. Williams et al. focused on hospice patients and carers and compared their interest in research with ambulatory senior citizens. The patients and carers showed slightly lower interest in research compared to senior citizens: in survey research, (45% and 57%) and in therapeutic research (46% and 60%) respectively [18].

Two other studies focused on a hospital setting. Daly et al. showed the willingness of newly diagnosed cancer patients (70%) to participate in psychosocial cancer research through the evaluation of a data registry to advance research [22]. Christ et al. described the establishment of a designated social work research unit in a major academic cancer research institution. They described the process of developing understanding and collaboration among clinicians and researchers, which required time and exposure to each other’s goals, values, skills and limitations [21].

#### Participation in research using surveys, interviews, observational or participatory methods

All except two studies [19,20] were evaluations of actual research experiences and were retrospectively addressing participation in research on EoL issues, which could involve sensitive issues. Most studies made use of questionnaires and qualitative interviews or both, and two of focus group discussions [19,32], of which one applied a participatory approach [32]. One study reported on feedback to an observational design [30].

These studies were carried out in a variety of settings and were all targeted at patients or their informal carers’ participation. In Kendall et al. a sample of researchers was included [28].

The studies all reported a greater number of positive than negative results. Where the two studies using a hypothetical approach enquired about preferences [19,20], the other studies either focused on patterns of recruitment to uncover participants’ views on research [24,27,30], or on the experiences of patients and carers taking part in research that used social science methodology, or both [24-27].

Barnett showed that the majority of patients found a one-hour long interview a positive, therapeutic experience [23]. In Davies et al. most patients (88%) and carers (96%) agreed to be interviewed, while 63% reported comfort from being interviewed and only 5% disliked the experience. But they reported that there was a 95% follow-up rate of the interviews [24]. In Emmanuel and Emmanuel, 87% of approached patients and 97% of carers were interviewed about death, dying and bereavement. 88% of patients and of 89% carers reported little or no stress from participating in an interview. The results on helpfulness were more equivocal with 16% of patients saying it was very helpful, 29% somewhat, 49% little or not, and 19% of carers saying it was very helpful, 34% somewhat, and 44% little or not [26].

Gysels et al. found differential patterns in decline and acceptance of interviews by patients with different conditions and across settings. Patients and carers were capable of deciding whether to participate in interviews and negotiate how they wanted this to happen [27]. Takesaka et al. reported that 22% experienced distress due to being interviewed about EoL issues [31]. Pessin et al. showed that 75% of patients did not experience psychosocial research as a burden, and 68% found it moderately to highly beneficial [29]. Wright et al. assessed the involvement of patients as co-researchers which did not cause them distress, however some logistical and methodological problems appeared [32]. A study from thanatology assessed risks and benefits associated with in-depth interviewing. Patients found discussing sensitive issues painful, but they said they benefitted by talking with a researcher and thought they could do this more openly than with a health professional. The researcher reflected on the impact of this research on herself, and recognized sadness but also meaningfulness [33]. In Kendall et al., people affected by cancer and researchers found that people nearing the EoL want to be offered the chance to participate in research, provided it is conducted sensitively [28]. Phipps et al. compared consenting participants and refusers in observational research and found that those participating thought they had more to gain from research compared with refusers (=0.04), and felt that pain was more of a problem (*p* < 0.001) [30]. Dobratz et al. explored issues and dilemmas related to non-participation, attrition, and needs for assistance in research with home hospice participants. They found that 28.8% required assistance because of physical impairments, and 14.1% were unable to participate or lost to the study because they suffered from cognitive disturbance, and physical distress. Those who completed testing expressed gratitude at being able to contribute information that they believed would benefit others [25].

One study that applied a hypothetical design explored cancer patients’ views and preferences on taking part in longitudinal questionnaire-based research studies. This study shows the importance of flexibility in planning interviews as fluctuating symptoms may affect the ability and willingness to participate in this population [20]. In a study using hypothetical questions, patients very near to death expressed a positive wish to participate in research [19].

#### Participation in clinical trials

Two studies were identified that considered research participation in clinical trials. One was targeted at patients with cancer and used a hypothetical approach [17] and one at hospice staff obtained empirical data [34].

White et al. found that patients were interested in participating in randomised controlled trials (RCTs) that concerned symptom control and relatives supported their participation in such trials. Over 75% of patients expressed altruistic views as a reason for participation. Many were prepared to complete short questionnaires, accept extra medications, investigations, hospital visits or admissions within a trial context. However deterrents were: concepts of ‘randomisation’, ‘placebo-control’ and ‘blinding’, and the possibility of side-effects [17].

Grbich et al. documented the impact of a trial on hospice staff which initially produced high levels of stress, but diminished over time. The stress was replaced by enthusiasm for the achievements of the study and sadness at the loss of benefits post-trial [34].

### Characteristics of participants and the study designs

The included studies revealed various characteristics that were either related to the individuals or groups investigated or were associated with the specific area or type of research.

#### Participant characteristics

Dobratz et al. provided insight into the problems commonly experienced by palliative care patients involved in research. Cognitive disturbance, and physical distress often played a role in attrition and in needing assistance to enable participation [25]. This same finding was made by [Shipman et al. 20](see above) . Phipps et al. compared ‘consenters’ with ‘refusers’ (see above), showing that consenters thought they had more to gain from research and felt that aches or pain were more of a problem for them [30].

Williams showed that young people were more interested in taking part in research than older participants, but there was no difference in attitudes between older hospice and non-hospice patients [18]. Increasing age also appeared in White et al. as the only negative factor indicative of less involvement in trial research [17].

Gysels et al. showed differences in motivations to research participation by patient groups, and between patients and informal carers [27]. Takesake et al. showed higher distress for women, younger carers and family members of patients with cancer. They caution however that although these associations were strong, the reasons for them are uncertain [31].

Emmanuel and Emmanuel identified individual characteristics of patients associated with the impact of a research interview. People with pain, those who are able to find more personal meaning in dying, and being less at ease with talking about dying, were significantly more likely to report more stress. Patients from ethnic minorities, those who were anxious about the end of life, and those who were more spiritual or serene were significantly more likely to find an interview helpful [26].

Terry et al. showed that patients very close to death provide unique understandings as death nears [19].

#### Study design characteristics

Cassarett et al. showed that hospice organisations were more supportive of research that they considered to be concerned with issues directly relevant to hospice care: pain management and referral to a hospice [16]. Shipman et al. enquired about the best format with which to conduct longitudinal studies using questionnaires. Patients preferred health professionals known to them to contact them about a study; they preferred face-to-face interviews at home but they were prepared to take part in them in hospital [20]. Face-to face interviews were also the preferred method by patients in the studies by [Daly et al. and Terry et al. 19,^22^].

There is also evidence on the feasibility of conducting interviews with carers and patients jointly or separately. The study by Davies et al. outlined the disadvantages of joint interviews [24], and Takesake et al. showed that joint interviewing did not cause distress in the study’s sample [31]. In three papers the benefit was shown of a researcher who was not involved in the daily care of a patient to whom patients could open up and give their genuine view on events [24,27,33]. However, patients experienced distress when asked to discuss death [26,29].

Grbich et al. showed the potential to improve the conduct of a large RCT by carrying out a parallel qualitative study [34].

## Discussion

### Willingness to participate in EoL research

All of the studies that evaluated willingness to participate in EoL care research showed positive outcomes across the different parties involved [15-20,22,25,28]. The majority of patients were willing to take part in research [15-20,22,25,28] and their informal carers were supportive of this [17,18]. Also hospice staff and hospice organizations were found to have positive attitudes towards participating in research [15,16]. The studies that reported on health professionals showed the most equivocal results [15,34].

The studies illuminate the factors that influence willingness to participate in research. Disability, physical distress and cognitive impairment formed barriers to individuals’ participation in research studies. However, some studies showed ways to enable patients to participate by providing assistance to complete questionnaires and to use flexible and responsive approaches to data collection to accommodate patients’ physical and cognitive abilities [25]. Participation for patients was often experienced as empowering and this was expressed through gratitude towards those who made it possible to contribute [25].

### Experiences of participating in EoL research

All 10 studies providing quantitative results showed that participating in research at the EoL was a positive experience for the majority of patients and their carers [20,23-27,29-32]. Benefits of participation were identified as being therapeutic or giving comfort, especially in interviews [23,24,26,27] which provided the opportunity to reciprocate for good care, and to contribute to service improvement. However, there was a minority who experienced involvement in research as distressing and this was related to either, characteristics of the participants, the type of research, or the way it was conducted.

The studies that evaluated actual experiences of research participation provided particularly useful insights on the benefits and risks of working with patients and their carers at the EoL and suggest future directions for research. The studies focusing on attitudes were helpful in identifying participants’ beliefs and values about research. Future attitude studies could widen their focus and explore how these values are informed and used. Studies that used hypothetical questions are important in difficult-to-research areas but one needs to be aware that they provide a normative view on reality. Most studies have approached patients with a set of hypothetical questions, particularly those involving trial research. Examining actual experiences as patients go through these trials – what motivated them, and the challenges encountered – is likely to shed a different light on this.

Age appeared to be a factor that influenced attitudes towards participation in research [17,18]. Differences were found in the motivation to participate by patient groups [27] and individual characteristics [26] and despite the strong associations found in quantitative studies there are no discernable explanations [31]. The identified patterns of views and impacts of research require exploration beyond the typical demographic variables, and designs are needed to capture the complexity of concerns and interactions between those involved in research. A way forward is the development of mixed-methods designs, which offer multiple opportunities to verify data and its interpretation. However, these designs require thorough planning from the outset and reflexivity in the way they are conducted. Palliative care encompasses those patients and carers at diagnosis through to death and beyond, and as such longitudinal studies could provide insight in to how levels of willingness and experiences with research change throughout these stages, and could help determine the best ways to conduct studies at different stages.

### Gaps in the evidence-base

We did not find any evidence of participation in research from beyond the US, the UK or Australia. This reflects the concentration of palliative care services and research centres in these countries [6]. Studies from beyond the developed world will add to the findings from this review and are expected to show how different environments and values impact on attitudes and experiences with research. This geographical bias also determined the study settings, which were mainly in-patient hospices or hospitals. Other settings such as the home and the community were under-represented.

The majority of the studies focused on patients with cancer, while there were only a few studies that included other advanced diseases. It is important to include the range of diagnoses that have palliative care needs as different patient groups can have distinct concerns regarding research. The gap in knowledge about cancer patients in the advanced stages of illness seems to be addressed since the review undertaken by [Todd et al. 12]. Most of the studies identified in this review targeted the later stages of illness as concerns regarding research were expected to become more pronounced nearer to death. There were studies that did not precisely describe the stage of illness which is necessary to compare experiences within the heterogeneous population who require palliative care. Given the findings regarding age as a significant influencing factor, future studies could include older people.

As the ethical discussion about research participation revolves for the most part around patient vulnerability, the studies included here have mostly investigated patient views. A variety of other stakeholders were represented, but more evidence is needed about the roles they fulfil and the particular contribution of each of these parties. The views of members of ethics committees or funders of EoL research were absent. Also studies that focus on interactions between different stakeholders could reveal perceived impacts of research, areas of conflict or opportunities for mutual understanding.

### Research participation as teamwork

Several studies showed that research at the EoL is best realised as a collaborative effort [21,24]. Division of tasks between researchers and clinicians can lead to frequent and extensive discussion that provides opportunities to develop a realistic appreciation for each other’s capabilities and limits [21]. Patients and other participants in research need to be engaged as early as possible [15,35]. This requires a campaign of public education [32] regarding the importance of research [36]. Health professionals concerns also need to be addressed [15,21,34,37,38]. Conditions conducive to research are required, including: the sharing of skills, supportive institutions [28,34,39], formal partnerships between care providers and academic entities and extra resources [32,34,38]. Flexibility is required in the policies, protocols [34] and evaluations made by ethics committees in order to counter administrative constraints and protective attitudes that often hinder research [40,41]. Research in palliative care often requires mixed methods or a diversion from standard procedures (for example when dealing with informed consent), which are circumstances that ethics committees are generally not used to working with [40,41]. A ‘relational ethics’, where researchers and ethics committees work in partnership, has therefore been promoted as a suitable model to enable valid and sensitive research in palliative care [40,41]. Participatory designs were found particularly suitable to actively include a diversity of participants who can determine the design and conduct of research [28,30,34,39].

### Research with care

This review shows that the ethical concern about patients at the EoL as too vulnerable to participate in research is often unjustified. However, the studies providing evidence about participation in research should not simply dismiss these concerns that have grown through debate and argumentation in ethics. Rather, they need to put these side by side and critically assess how the findings can inform the best way forward. Therefore, we analysed the rationale that underlies the reasoning about research in EoL care. We investigated the normative framework that organises the problematic issues pertaining to EoL care research from the entire body of evidence. The resulting conceptual model frames our understanding about what research is and does in EoL care settings. The model shows the ‘taken for granted’ knowledge on which respondents base their actions and develop their attitudes.

**Figure 2:**
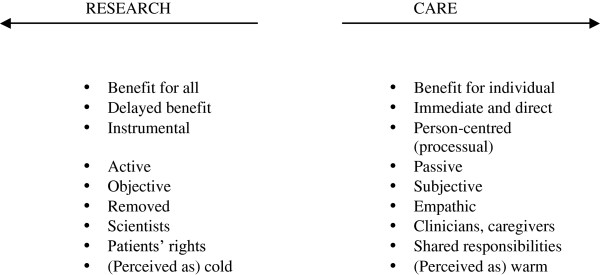
Oppositional values of research and care at the EoL.

The ethical arguments frame research and care as if they have oppositional goals and values (see Figure 2). This is what is called the deontological-utilitarian conflict where the goal of enhancing outcomes for all people needs to be balanced against the goal of ensuring optimal comfort and benefit [42]. People should in other words not be subjected to greater risk than what they would endure in routine assessment or treatment. Research is understood as instrumental, using the patient and others taking part in it, as a means to an end. Care, on the other hand, has the best interests of the patient and others in mind. Research is for the benefit of future patients, as it is argued that patients will already have died by the time that the findings will have materialised in interventions, while care can be provided immediately and directly. Within this discourse, research is perceived as active, and those who participate in it as passive, as the latter are presented as being subjected to research. It is objective, and removed, while care is personal and close. Stakeholders representing different territories should stay on their own sides to avoid misunderstandings. Researchers who transgress these boundaries of research and care – for example, those who collect data through qualitative methods, which inevitably involves close communication and empathy, are told to take all precautions to protect patients from deception. In the same way, the clinician-scientist is challenged as inappropriate, as this could lead to coercion based on the trust developed between the health professional and the person being cared for. The opposition is reflected in the metaphors attached to these domains where positions are crystallised in terms of ‘warm’ care and ‘cold’ research. 

Where objections to research were expressed in the studies under review, these came from the concern that research would detrimentally affect the care that patients need. For example, the protective attitudes by hospice staff towards their patients in [Kirsch et al. 15] gave evidence of such a concern, or the apprehension patients expressed towards terms associated with trial research in [White et al. 17], where terms such as ‘randomisation’, ‘placebo-control’ and ‘blinding’ deterred patients from participating in research. This reflects similar concerns found in an earlier study of patients’ and the public’s view of the process of randomisation, where the role of chance by using computerised procedures – equivalent to tossing a coin – were used as a way of determining treatment allocation in the context of life-threatening research, and were found to be upsetting and trivialising [43].

**Figure 3:**
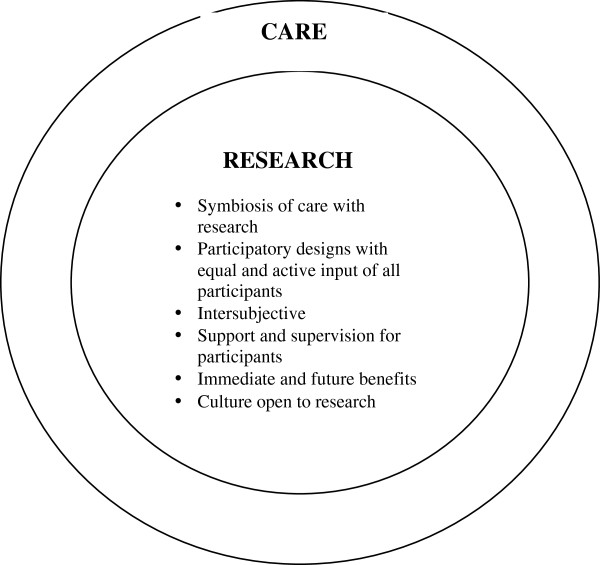
Research with care.

Successful studies were the result of research that was conducted with care, with for example tailored data collection to accommodate patients’ disabilities and to enable their participation [25]. The studies using a participatory design were also based on mutual recognition of each other’s strengths and weaknesses and were conducted within a teamwork-based setting [17]. Such studies suggest that a different model of research in EoL care is needed (see Figure 3). When research is embraced by care, many of the ethical objections become invalid. The issue of being used as a means to an end, or in other words as a mere instrument for research goals, does not apply any longer when research is embraced by care. A collaborative model with equal input of all the parties involved in research, who share a concern to care will lead to sensitivity to the needs that arise during the conduct of research studies. This also guarantees the immediate benefits that involvement holds for patients, such as empowerment, self-validation or the opportunity it offers for altruism [13,19]. A relational approach where the ideas of different stakeholders from a diversity of disciplines are exchanged, can lead to an intersubjective view on what is investigated and the way research is conducted. The concern about granting the right to participate in research shifts towards mutual responsibility among those involved and is realised through open dialogue. The attention then focuses on what support and supervision is needed in order to optimise participants’ contributions [8,17] and this can lead to a change in culture in terms of attitudes towards research [21,34]. 

### Limitations

This paper set out to systematically assess the evidence relating to barriers and solutions to participation in research at the EoL. However the review is limited as only original research studies were included. Papers presenting ethical debates on participation within EoL care research were excluded. For a comprehensive overview of the field, the ethical arguments need to be read alongside this review. It is also possible that the gaps identified in the evidence-base are not neutral with regard to the aim of the study, and that they represent an indication for undetected problems in EoL research, which can lead to publication bias.

## Conclusion

This review provides an evidence-base about ethical concerns regarding research at the EoL. Although the literature focused most on patients with cancer, a range of other participant groups were also represented, such as health professionals, carers and researchers. The evidence shows that all those involved in research are in principle supportive of research at the EoL. The group most likely to have concerns about involving patients at the EoL were health professionals.

This review showed that the ethical concerns regarding patient participation in EoL care research are often not justified. Patients are generally willing to contribute to research and the majority of those who take part in research, report it as a positive experience. Patients confirm that it brings them direct benefits. However, there was a minority of people for whom taking part caused distress, requiring increased sensitivity to individuals’ changing needs and circumstances. Assistance needs to be in place for such situations. The palliative care population commonly experience fluctuating symptoms across their disease trajectory, which can cause varying levels of physical and cognitive impairment. Therefore, research needs to be flexible and equipped with methods that can enable patients and carers to participate in research on EoL care.

Denying patients the opportunity to participate in research on the basis of an a priori label of vulnerability is paternalistic. Given the diversity of this population, patients need to be approached as individuals, to assess whether it is desirable to involve them in research. This makes the model presented here not only applicable to the EoL population, but to all potentially vulnerable people.

## Competing interests

The authors declare that they have no competing interests.

## Authors’ contributions

IJH identified the need to review this area of research and is PI for project MORECare, the MRC project, which this review is part of. All authors consented to study design. MG undertook the review and drafted the paper with significant input from CE and IJH. All authors approved the final version.

## Pre-publication history

The pre-publication history for this paper can be accessed here:

http://www.biomedcentral.com/1471-2288/12/123/prepub
